# An Ensemble Deep Convolutional Neural Network Model with Improved D-S Evidence Fusion for Bearing Fault Diagnosis

**DOI:** 10.3390/s17081729

**Published:** 2017-07-28

**Authors:** Shaobo Li, Guokai Liu, Xianghong Tang, Jianguang Lu, Jianjun Hu

**Affiliations:** 1 School of Mechanical Engineering, Guizhou University, Guiyang 550025, China; lishaobo@gzu.edu.cn (S.L.); xhtang@gzu.edu.cn (X.T.); jglu@gzu.edu.cn (J.L.); 2 Key Laboratory of Advanced Manufacturing Technology, Ministry of Education, Guizhou University, Guiyang 550025, China; guokai_liu@163.com; 3 Department of Computer Science and Engineering, University of South Carolina, Columbia, SC 29208, USA

**Keywords:** bearing fault diagnosis, D-S evidence theory, convolutional neural networks, deep learning

## Abstract

Intelligent machine health monitoring and fault diagnosis are becoming increasingly important for modern manufacturing industries. Current fault diagnosis approaches mostly depend on expert-designed features for building prediction models. In this paper, we proposed IDSCNN, a novel bearing fault diagnosis algorithm based on ensemble deep convolutional neural networks and an improved Dempster–Shafer theory based evidence fusion. The convolutional neural networks take the root mean square (RMS) maps from the FFT (Fast Fourier Transformation) features of the vibration signals from two sensors as inputs. The improved D-S evidence theory is implemented via distance matrix from evidences and modified Gini Index. Extensive evaluations of the IDSCNN on the Case Western Reserve Dataset showed that our IDSCNN algorithm can achieve better fault diagnosis performance than existing machine learning methods by fusing complementary or conflicting evidences from different models and sensors and adapting to different load conditions.

## 1. Introduction

As one of the core components of rotating machinery, rolling element bearings are used to constrain relative motions to only the desired motion and reduce friction between moving parts. Bearings are always expected to work 24 h per day in actual production. Any failure with the bearings may lead to unexpected consequence of the whole machine. As bearing failures always bring downtime, expensive repair and hidden cost to enterprises, real time monitoring and precise fault diagnosis are critical to avoid catastrophic damages.

In the past decades, a variety of methods have been developed in bearing fault diagnosis, such as vibration analysis [1], acoustic analysis [2], noise analysis [3], thermal imaging analysis [4] and so on, among which the vibration analysis has proven to be the most efficient [5]. Many vibration signal processing tools have been used in signal preprocessing such as Fourier spectral analysis [6], wavelet analysis [7], empirical mode decomposition [8], and multi-wavelet transformation [9]. These vibration analysis methods have achieved good performance from non-adaptive analysis to adaptive analysis and from qualitative analysis to quantitative analysis [10]. However, good performance with these methods highly depends on the expert experience and knowledge.

In addition to the application of diverse signal preprocessing approaches, many artificial intelligence methods have been applied to features extraction and classification, two main steps in bearing fault diagnosis. Time domain statistical analysis, multi-wavelet transformation and fast Fourier transformation are always used for features extraction. Feature selection methods such as principal component analysis (PCA) [11] and independent component analysis (ICA) [12] are employed to select most useful features. Classifiers such as Bayes Classifier [13], k-nearest neighbor (KNN) [14], Multi-layer Perceptron neural networks (MLP) [15], support vector machines (SVM) [16], Decision Trees (DT) [17], and Random Forests (RF) [18] have all been applied to bearing fault diagnosis. Among them, MLP is known for its capability to learn features with complex and nonlinear patterns. It has also been reported that SVM is superior to the other AI algorithms in fault diagnosis due its memory efficiency and good performance when the number of features is greater than that of samples [19,20,21].

Recently, deep learning methods have received amazing success in pattern recognition and machine learning application domains due to their outstanding capability to learn complex and robust representations. Many classification problems have been successfully solved by various deep learning models such as deep belief network [22], deep Boltzmann machine [23], deep auto encoder [24], convolutional neural network (CNN) [25] and so on. Deep learning has also been applied to fault diagnosis recently. Guo [26] proposed a hierarchical adaptive deep convolution neural network (ADCNN) on bearing fault analysis, which first output the fault type and then analyze the fault size. Chen et al. [27] proposed a 2D-CNN model for gearbox fault diagnosis through combining time domain statistical features with 256 statistical frequency domain root mean squared (RMS) values. An 1D CNN model based on the raw time series data was proposed for motor fault detection by Ince et al. [28]. Tran et al. [29] developed a deep belief model for reciprocating compressor fault analysis.

Although the deep learning methods have achieved great success in fault diagnosis, there still exist two potential issues. First, selecting appropriate network structures, parameters, and algorithms is critical to the success of deep learning approaches. Second, the signal data in all current deep learning methods for fault diagnosis are from a single sensor, which may not provide reliable information in terrible working environments. The adaptability and the anti-interference ability of different sensors varies widely so prediction models based on single source of information could lead to misdiagnosis. It is desirable to exploit multi-sensor information source for more reliable fault diagnosis by effective information fusion approach, which is complicated by the fact that different sensor locations and sensor qualities.

In this paper, we propose a novel algorithm that integrates deep neural networks with multiple sensor information with an improved Dempster–Shafer (D-S) evidence theory [30] based data fusion model for bearing fault diagnosis. Our fusion model can combine multiple uncertain evidences and give the fusion result through merging consensus information and excluding conflicting information. In recent years, D-S evidence theory and its variants have been widely used in multi-sensors data fusion, decision analysis, fault detection and other industrial fields. However, two defects in traditional D-S evidence theory still exist, which we addressed in our proposed fusion model: one is evaluating the basic function of the evidence body objectively; and the other is solving the conflict evidences coming from different sources.

The rest of this paper is structured as follows. Section 2 introduces our improved D-S evidence theory (IDS) through calculating the similarity among different evidences and assigning the weights to the original evidences by the modified Gini index. Section 3 presents the proposed IDSCNN fault diagnosis model with detailed description. We trained several CNN models with different structures and parameters, which are synthesized by our IDS method. Section 4 describes the experiment setup and presents the evaluation results on two sets of experiments that prove the superiority of the improved fusion model and the IDSCNN fault diagnosis algorithm. A conclusion is presented at the end of this paper.

## 2. Materials and Methods

### 2.1. Improved D-S Evidence Theory for Information Fusion

D-S Evidence Theory is a mathematical theory and general framework for reasoning with uncertainty, which allows one to combine multiple (usually conflicting) evidences from different sources and arrive at a degree of belief (represented by a mathematical object called belief function) that takes into account all the available evidence. D-S evidence theory has been shown [31,32] to achieve better performance in data fusion based classification compared to the traditional probability theory due to its capability to grasp the unknown and uncertainty. This method has been widely used in many fields in recent years such as machine health monitoring and fault diagnosis [33,34], engineering design [35], security defense [36], target recognition and tracking [36,37], decision-making [38], and information fusion [37,39]. A simple integration method [34] such as voting (used in LIBSVM (a library for Support Vector Machines) for multi-classification problem) has proven to have a relatively poor performance in bearing fault diagnosis compared to using the D-S evidence theory. Given all that, we chose the D-S evidence theory for model fusion in this paper.

#### 2.1.1. Preliminaries

D-S evidence theory assumes a finite set of elements $\Theta =\left\{A_{1},A_{2},\ldots ,A_{n}\right\}$, which is called the frame of discernment. The symbol m is a measure on the subsets of Θ and is called a basic probability assignment function (BPAF). This function is subject to the following qualifications:

(1) $$\begin{array}{c} m(\phi )=0\\ 0\leq m(A)\leq 1,\;\forall A\subset \Theta \\ \Sigma_{A\subset \Theta }m\left(A\right)=1 \end{array}$$

D-S evidence theory provides a very useful synthesis formula, which can combine evidence from different evidence sources. For A⊂Θ, in the frame of discernment Θ, there are finite basic probability assignment functions $m_{1},m_{2},\cdots m_{n}$, the synthesis formula is defined as the following:

(2) $$\left(m_{1}⊕m_{2}\ldots ⊕m_{n}\right)\left(A\right)=\frac{1}{1-k}\underset{A_{1}\cap A_{2}\ldots \cap A_{n}=A}{\sum }m_{1}(A_{1})\cdot m_{2}(A_{2})\cdots m_{n}(A_{n})$$

where

(3) $$k=\underset{A_{1}\cap A_{2}\ldots \cap A_{n}=\phi }{\sum }m_{1}(A_{1})\cdot m_{2}(A_{2})\cdots m_{n}(A_{n})=1-\underset{A_{1}\cap A_{2}\ldots \cap A_{n}\neq \phi }{\sum }m_{1}(A_{1})\cdot m_{2}(A_{2})\cdots m_{n}(A_{n})$$

*k* represents the degree of conflicting evidence and the coefficient 1/(1 − *k*) is called the normalization factor which ensures that the sum of BPAs can be unit.

The traditional D-S evidence theory presents a good method for evidence fusion. However, some limitations still exist, which can lead to failure on evidence fusion under certain circumstances such as some complex practical environment with probable conflicts of different evidence. Many researches have been done to solve the evidence paradox issue. Table 1 shows four common paradoxes [40], namely complete conflict paradox, 0 trust paradox, 1 trust paradox and high conflict paradox, which cause fusion difficulty for traditional D-S theory. It should be noted that, in Table 1 and Table 4, propositions *A*, *B*, *C*, *D*, *E* and Θ are the elements of the frame of discernment, which contains all the categories and can be interpreted as the bearing fault types in our problem.

In the complete conflict paradox, the conflict factor k can be calculated as *k* = 1, which causes the denominator of Formula (2) to become zero when we apply Formula (3) to the two evidences *m*_1_ and *m*_2_. Though evidences *m*_1_, *m*_3_ and *m*_4_ support the proposition, the D-S combination rule cannot be used to synthesize the evidences under this circumstance.

For 0 trust paradox, the total conflict factor *k* can be calculated as *k* = 0.99 and the BPAs (basic probability assignment) can be obtained as *m(A)* = 0, *m(B)* = 0.727, *m(C)* = 0.273 according to Formulas (2) and (3). Though evidences *m*_1_, *m*_2_, and *m*_4_ support the proposition *A*, *m*_2_ = 0 totally negates this proposition. Under this circumstance, the BPA for proposition *A* will always be 0 no matter how many and how strong other evidences support *A*.

For 1 trust paradox, the total conflict factor can be calculated as *k* = 0.9998 and the synthesis results for propositions *A*, *B* and *C* is *m(A)* = 0, *m(B)* = 1, and *m(C)* = 0. Though all evidences support the proposition *B* with small BPAs, the synthesis result totally believes the proposition *B* is the right answer and denies other propositions which is contrary to common senses.

For high conflict paradox, the total conflict factor can be calculated as *k* = 0.9999 and the synthesis results are *m(A)* = 0, *m(B)* = 0.3571, *m(C)* = 0.4286, *m(D)* = 0, and *m(E)* = 0.2143. Though evidences *m*_1_, *m*_3_, *m*_4_, and *m*_5_ support proposition *A* with large BPAs, the synthesis results totally deny it due the high conflicts among evidences.

To address these issues, we proposed an improved D-S evidence theory through Euclidean distance matrix and modified Gini index, as stated below.

#### 2.1.2. The Improved D-S Evidence Theory

In this section, we will firstly calculate the similarity matrix through a Euclidean distance function. Then, we combine the similarity matrix with a modified Gini index to get the evidence credibility. We use the evidence credibility as weights to modify the original evidence and decrease the conflict evidence.

Define $\varepsilon_{i}$ as the creditable factor of the evidence $E_{i}$, then all the creditable factors form the creditable vector for the evidence set can be written as $\varepsilon_{i}=(\varepsilon_{1}\cdots \varepsilon_{2}\cdots \varepsilon_{n}),\;\varepsilon_{i}\in \left(0,1\right]$. Define BPA matrix $B_{nxN}$ (which consists of evidence $m_{i}\left(i=1,2,\cdots ,n\right)$), n stands for the number of evidence and *N* stands for the number of propositions in the frame of discernment Θ. Thus, $B_{ij}=m_{i}\left(A_{j}\right)$ stands for the *j_th_* BPA value of the *i_th_* evidence. Define $p_{i}=\left(m_{i}\left(A_{1}\right),m_{i}\left(A_{2}\right),\cdots m_{i}\left(A_{n}\right)\right)$ as the *i_th_* row in BPA matrix $B_{nxN}$, then the vector $║p_{i}-p_{j}║$ is the Euclidean distance between $p_{i}$ and $p_{j}$ which stands for the similarity between $E_{i}$ and $E_{j}$.

Note

(4) $$d_{ij}={║p_{i}-p_{j}║}_{1}=\sum_{k=1}^{N}\left|m_{i}\left(A_{k}\right)-m_{j}\left(A_{k}\right)\right|$$

Thus, we can get a distance matrix

(5) $$D=\left[\begin{array}{ccccc} 0 & d_{12} & d_{12} & \cdots & d_{1n}\\ d_{21} & 0 & d_{23} & \cdots & d_{2n}\\ \vdots & \vdots & \vdots & \ddots & \vdots \\ d_{n1} & d_{n2} & d_{n3} & \cdots & 0 \end{array}\right]$$

In Equation (4), $0\leq m_{i}\left(A_{j}\right)\leq 1\;and\;\Sigma_{j=1}^{N}m_{i}\left(A_{j}\right)=1$, so the maximum value of $d_{ij}$ is $\max\left(d_{ij}\right)=2$.

We define the regularized element for the following evidence credibility as below:

(6) $${\tilde{d}}_{ij}=\frac{d_{ij}}{4}+0.5,\;{\tilde{d}}_{ij}\in [0.5,\;1]$$

**Figure 1:**
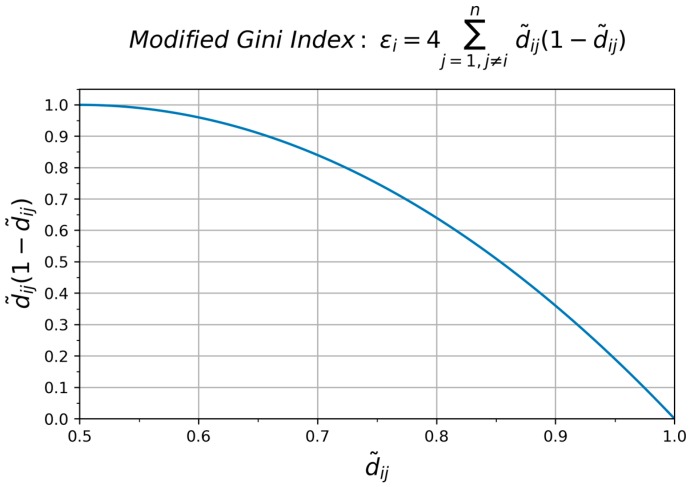
The modified Gini Index curve.

Credibility factor $\varepsilon_{i}$ reflects the deviation degree among evidence set $E_{i}$. This means $m_{i}\;$is consistent with other evidence when its credibility factor $\varepsilon_{i}$ is relatively large and close to 1 while $\varepsilon_{i}$ is singular compared with others when its value is a relatively small value close to 0. Thus, $\varepsilon_{i}$ should be a decreasing function ${\tilde{d}}_{ij}$ such that $\varepsilon_{i}=\Sigma_{j=1}^{n}f\left({\tilde{d}}_{ij}\right)$. Here, we employ the modified Gini Index $\varepsilon_{i}=4\Sigma_{j=1,j\neq i}^{n}{\tilde{d}}_{ij}\left(1-{\tilde{d}}_{ij}\right)$ as our decreasing function that satisfies our requirements. Within the argument range [0.5, 1], $\varepsilon_{i}$ is a decreasing function. Credibility factor $\varepsilon_{i}$ increases to 1 when the distance $d_{ij}$ between two evidences decreases otherwise $\varepsilon_{i}$ decreases to 0 (Figure 1).

The regularized evidence credibility is

(7) $$\varepsilon_{i}^{r}=\varepsilon_{i}/\Sigma_{i=1}^{n}\varepsilon_{i}$$

The credibility factor $\varepsilon_{i}$ reflects the similarity degree of certain evidence with others. The creditable factor $\varepsilon_{i}^{r}$ is a normalized result. When the similarity between certain evidence and others increases, the creditable factor $\varepsilon_{i}^{r}$ increases and vice versa.

After we get the creditable factor $\varepsilon_{i}^{r}$, we can correct the raw evidence. Define the $m_{i}\left(A_{j}\right)$ as the *j_th_* proposition of *i_th_* evidence of the raw evidence and $m_{i}^{*}\left(A_{j}\right)$ as the rectified BPA, then

(8) $$\{\begin{array}{c} m_{i}^{*}\left(A_{j}\right)=\Sigma_{i=1}^{n}m_{i}\left(A_{j}\right)\varepsilon_{i}^{r}\\ m^{*}\left(\phi \right)=0 \end{array}\;\forall A\in \Theta ,A_{j}\neq \phi$$

Because $m_{i}\left(A_{j}\right)\in \left[0,1\right]$ and $\varepsilon_{i}^{r}\left(A\right)\in \left[0,1\right]$, the modified evidences satisfy $\Sigma_{A\subset \Theta }m^{*}\left(A\right)\leq 1$, we scaled the above result though the following expression

(9) $$m^{\prime }\left(A\right)=\left[m^{*}\left(A\right)-\min\left(m^{*}\left(A\right)\right)\right]/\left[\max\left(m^{*}\left(A\right)\right)-\min\left(m^{*}\left(A\right)\right)\right]$$

It should be noted that we introduce the end elimination mechanism here and set the minimum BPA to zero through the above expression. This will enhance the BPAs of other propositions intuitively and give higher confidence to the survived propositions. In fault diagnosis, we prefer the diagnosis result with higher probability intuitively.

The above results are normalized for the comparison in the latter validation within the same range. The decision rule for the improved D-S evidence theory compares the modified BPAs and selects the fault proposition with the maximum BPA evidence as the fusion result.

In summary, there are five steps in our improved D-S evidence theory for evidence fusion:

(1) Calculate the distances matrix ***D*** and its elements $d_{ij}$ among raw evidences.
(2) Calculate the evidence credibility $\varepsilon_{i}\;$using the modified Gini Index expression.
(3) Calculate the probability of weighted proposition m*(A).
(4) Calculate the final evidence $m^{\prime }\left(A\right)$ through scaling and normalizing for validation.
(5) Calculate the maximum $m^{\prime }\left(A\right)$ and select the relevant proposition as the diagnosis result.

### 2.2. The IDSCNN Ensemble CNN Model for Bearing Fault Diagnosis

**Figure 2:**
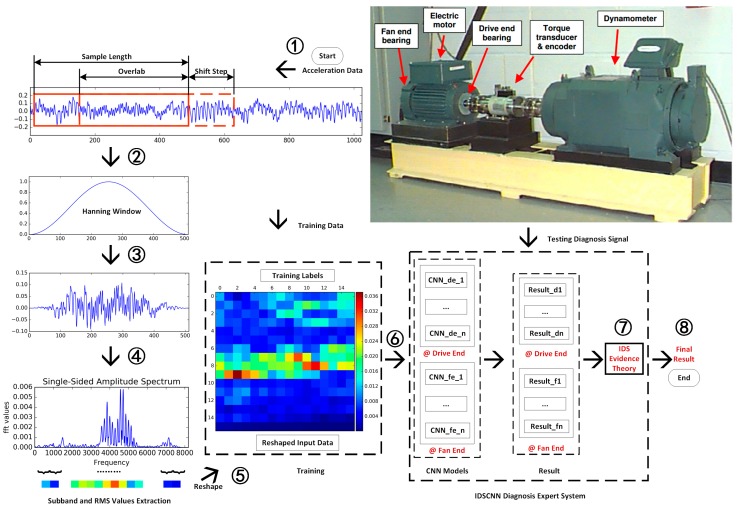
Flowchart of the IDSCNN based fault diagnosis.

There are three steps in our IDSCNN diagnosis model: Data preparation, Model Training, and Model testing, as shown in Figure 2.

#### 2.2.1. Data Preparation

Data acquisition and preprocessing are needed to train our CNN models, which is described as Blocks 1–5 in Figure 2. The raw signals for our bearing fault experiments are accelerator vibration signals from two sensors. For a given raw accelerator signal, we use a sliding window of size 512 with shift step size 200 to scan the signal and generate the raw data samples. Thus, for any two consecutive samples, there will be an overlap of 300 data points.

Feature extraction from raw sensor data is critical in machine monitoring. As shown in Figure 1, we use root mean square (RMS) over a sub band of the frequency spectrum as feature for our CNN model, which has the advantage of maintaining the energy shape at the spectrum peaks [27,41]. Our sampling length $N_{s}$ is set as 500 as suggested by Wade [42], who did a benchmark study on the CWRU data with trial and error and suggested that $N_{s}=500$ for the 12 k data. To avoid spectrum leakage, we multiply the selected time domain signal with a Hanning window and then obtain the FFT spectrum. We use the fft size (number of Fourier coefficients) $N_{\mathrm{fft}}=16,384$ and get the relevant frequency spectrum. Since the frequency spectrum is symmetrical, we only take the single-sided data for constructing the RMS maps as the input data for training CNN models. The sub band length-*b* for RMS values will be changed according to the size of RMS maps. Each sub band should have the same length except the last one if *N*/2 cannot be divided without remainder by the RMS map size. For example, if the size of RMS map is 32 × 32, *b* = 8192/(32 × 32) = 8 and if the size of RMS is 16 × 16, b = 8192/(16 × 16) = 32. For convenience of calculation, we take two kinds of RMS map sizes (16 × 16 × 1 and 32 × 32) as the size of our training data.

#### 2.2.2. The IDSCNN Model based on CNNs

**Figure 3:**
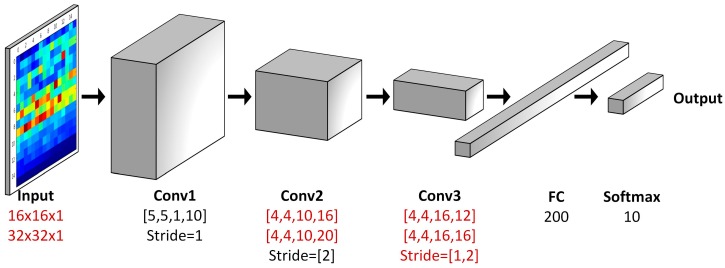
The convolution neural network model used in IDSCNN.

As shown in Figure 2, our IDSCNN prediction model is composed of an ensemble of CNN classifiers trained with two sensor signals, whose outputs are fused using the improved D-S fusion algorithm. Convolutional neural networks are selected here due to their capability to learning hierarchical representations. The structure of our CNN models is shown in Figure 3. It is composed of three convolutional layers plus a full connection layer. To explore the effect of parameters on the prediction performance, we have evaluated different parameter configurations as shown in the Table 2. There are two choices for the input size (16 × 16 × 1 or 32 × 32 × 1), convolutional layer 2 ([4,4,10,16] or [4,4,10,20]), convolutional layer 3 ([4,4,16,12] or [4,4,16,16]) and strides ((1,2,1) or (1,2,2)) for the three convolutional layers, respectively. We implemented the CNN models using Google Tensorflow 1.2.0rc2. All experiments are conducted on a computer equipped with a NVIDIA GPU 960M. We take the Adam stochastic optimization algorithm as the training algorithm due to its good performance, computational efficiency and memory-saving.

#### 2.2.3. Model Testing

To investigate IDSCNN performance, we conduct three different ways to build the IDSCNN models: (1) we combine all the diagnosis results from CNN models trained with signals from the drive end sensor; (2) we combine all the diagnosis results from CNN models trained with signal from the fan end sensor; and (3) we combine all the results from CNN models trained with signals from both sensors. The results of the combinations will be discussed in section.

### 2.3. Experiment Set-Up

**Figure 4:**
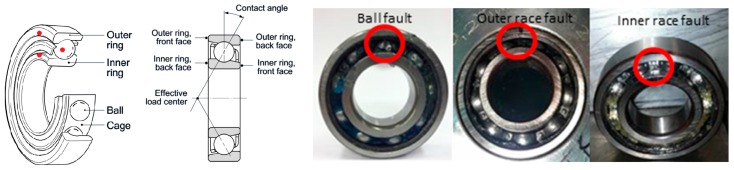
Bearing Structures and Fault Location Sketch Map.

To facilitate experiment verification and performance comparison with other related research, we evaluate our diagnosis models over the widely used bearing data from the Case Western Reserve University (CWRU) Bearing Data Center [42,43]. There are four bearing fault types included in the datasets (Figure 4): normal, inner race fault, outer race fault, and ball fault. Single point faults were introduced to the test bearings using the electric discharge machine (EDM). The fault datasets are further categorized by the fault size (0.007 inch, 0.014 inch, 0.021 inch). The test stand is shown in the upper right corner of Figure 2. It consists of a motor (left), a torque transducer/encoder (center), a dynamometer (right), and control electronics (not shown). Vibration data were collected using two accelerometers. At both the drive end and fan end of the motor housing, the accelerometers were attached to the housing with magnetic bases at the 6 o’clock position. All the normal baseline data were collected at 48 k samples/second while all fault bearing data were collected at 12,000 samples/second. Vibration data were recorded for motor loads of 0 to 3 horsepower (motor speeds of 1797 to 1720 RPM).

Thus, we can get ten fault conditions for each load. The most common way for evaluating a deep learning model is using the training data for modeling and testing the performance of the model through the test data. In order to get an objective result, the test data should not appear in the training data; otherwise, the test result will be overly optimistic. Thus, we apply random uniform sampling to the original accelerator dataset. As shown in Table 3, 10,000 training samples and 2500 testing samples (1000 training and 250 test data for each fault condition) are picked for each load condition and generate Datasets A, B, and C, respectively.

## 3. Results

In this section, we present two sets of experiment results. In experiment set 1, we evaluate how well our improved D-S (IDS) evidence fusion model addresses the paradoxes compared with several existing fusion methods. In experiment set 2, we evaluate the performance of IDSCNN for bearing fault diagnosis and compare its performance with other methods.

### 3.1. Evaluation of the Improved D-S Evidence (IDS) Fusion Algorithm

To compare the IDS method with existing evidence fusion methods, we take four paradoxes described in Section 3.1 as examples. Evidences can be divided into consistent evidences and conflict evidences. The former type supports the same proposition and the latter type disagrees with other evidences. From Table 1, we can see that *m*_1_, *m*_3_, and *m*_4_ in complete conflict paradox; *m*_1_, *m*_2_, and *m*_4_ in 0 trust paradox; *m*_2_, *m*_3_, and *m*_4_ in 1 trust paradox; and *m*_1_, *m*_3_, *m*_4_, and *m*_5_ in high conflict paradox are all consistent evidences in the four paradoxes groups, respectively. The remaining propositions belong to conflict evidences. Traditional D-S evidence has been proved to fail in dealing with these four common paradoxes. Here we take four modified D-S evidence fusion algorithms from Yager [44], Sun [45], Murphy [46] and Deng [47] for comprehensive analysis. The fusion results are presented in Table 4.

In Table 4, we can see that both Yager and Sun solve the conflicts through allotting the conflict factor to the unknown proposition in Θ, which however increases the uncertainty. Yager’s method fails in the four conditions and cannot solve the paradoxes when the number of evidences is more than two. Under the four paradoxical circumstances, Yager and Sun fail in getting reasonable results due to the high uncertainty with unknown propositions in Θ. The synthesis results from Murphy, Deng and our IDS method have achieved good performances and relatively rational results. Overall, our IDS fusion method has achieved the best results for three paradoxes out of four and achieved the second best result for the remaining paradox.

In the following diagnosis experiments, we choose the proposition with the maximum BPA as the fusion result. Thus, for the maximum BPA, the closer to 1 the more confidence it presents intuitively. As shown in Table 4, our IDS method has the largest maximum BPA of 0.9284 in complete conflict paradox, 0.7418 in 0 trust paradox, 0.9406 in 1 trust paradox and is ranked second in high conflict paradox with BPA 0.6210. In general, our proposed IDS achieves good performance in dealing with the paradox evidences for fault diagnosis.

### 3.2. Evaluation of IDSCNN for Bearing Fault Diagnosis on the CWRU Dataset

**Figure 5:**
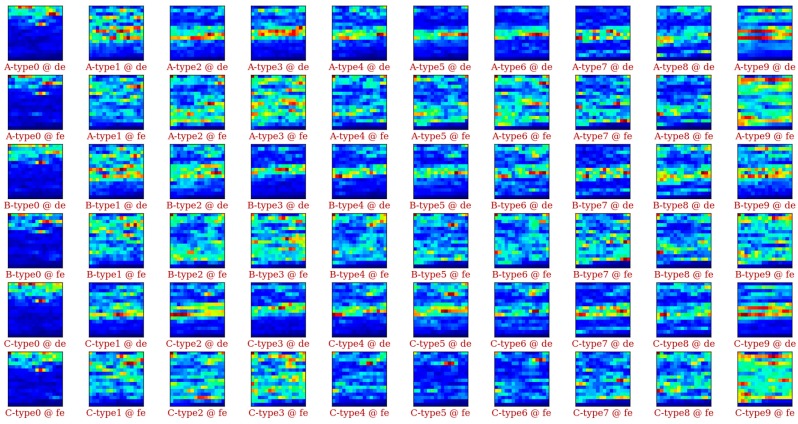
Data visualization of the input data 16 × 16 RMS maps.

As described before, the CRWU bearing datasets are acquired with different loading levels. To check how the loading level affects the vibration signals, we randomly extract one sample for different fault types (0, 1, ..., 9) and load condition. We then plot the 16 × 16 input data from different Datasets A, B, and C. In Figure 5, we can find that the RMS maps for a given fault type under different loads share significant similarities (column-vise similarity) in the CRWU bearing dataset. The RMS maps, however, vary significantly from fault type to fault type. Furthermore, even under the same fault state with the same load, there is a big difference between the drive end RMS map and the fan end RMS map. This means different sensors will carry different information. Combining different sensor information should give more information for fault diagnosis. Since we fix the length of the frequency spectrum, the 16 × 16 and 32 × 32 RMS maps are actually coming from the same frequency spectrum, but the 32 × 32 RMS maps carry more information than the 16 × 16 RMS maps.

First, we conduct experiments to evaluate the performances of our individual CNN models. In actual fault diagnosis scenario, the bearing load changes at all times. It is thus desirable that the fault prediction model can adapt to different loading conditions. To test this load adaptability of our models, we trained the CNN models on different training Datasets A, B and C corresponding to three different load conditions. We then tested their performance on testing Datasets A, B and C under three load conditions. There are nine combinations between training sets and testing sets, as shown in the top part of Table 5. For each model, we use the 10,000 samples for training and test it on the 2500 samples of different conditions. We should note that A, B and C now stand for different datasets for training and testing in the subsequent sections. 

As described in Table 2, we have developed 16 CNN architectures to evaluate. We build a model for each of these architectures and each sensor. Thus, we built 32 CNN models in total. To avoid random sampling errors, we repeated the same modeling process 20 times and took the average values as the final results, as shown in Table 5.

In Table 5, we can see that all CNN models have almost perfect performances on their training data and their performance vary on other testing datasets. Comparing the average accuracy (AVG) of the 32 CNN models, we can find some interesting observations. First, we can find that model #2 is the best model with accuracy of 97.89%. However, its adaptability from training set C to testing set A is 89.81%, which is lower than that of model #4, #9, #11 and #13. On the other hand, model #27 might be the worst model since its AVG is only 89.11%, but its local adaptability from training set B to testing set A (91.93%) are almost better than all CNN models with 32 × 32 input at the fan end. This phenomenon tells us that even the best selected model may have poor performance on certain circumstances and even the worst model may present relatively good performance under some conditions.

**Figure 6:**
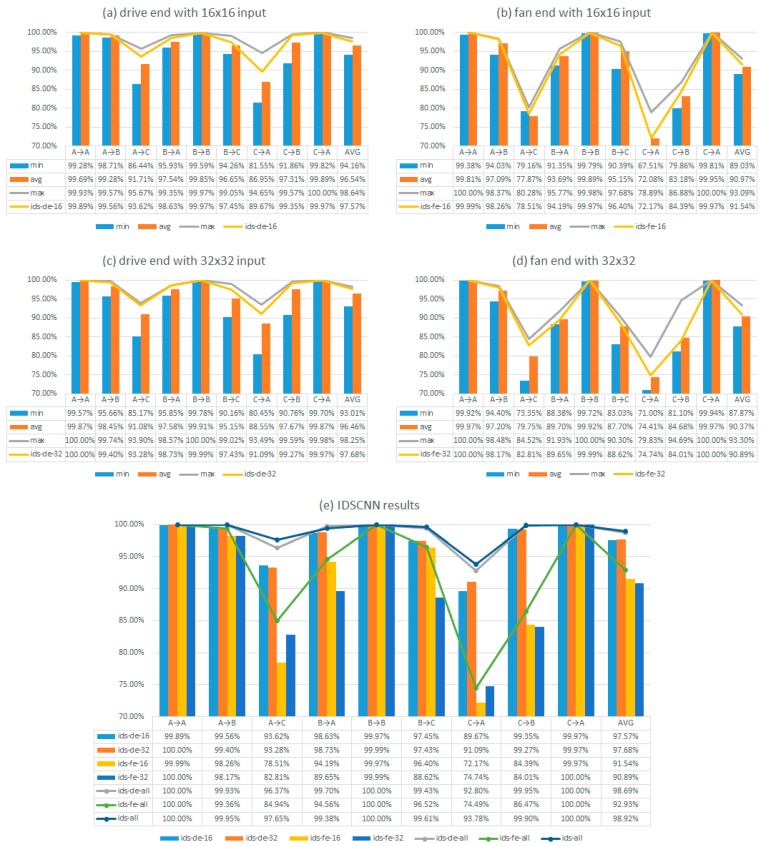
Results of the CNN Models and Proposed IDSCNN: (**a**–**d**) the results of individual CNNs trained with drive/fan end sensor signals and the IDS fusion results; and (**e**) IDSCNN results from fusion models with different component CNN models including fusing all 16 drive-end CNN models (ids-de-16, ids-de-32, and ids-de-all), fusing all 16 fan-end CNN models (ids-fe-16, ids-fe-32, and ids-fe-all), and fusing all models (ids-all).

We also compared the performance of models trained with driver-end and fan-end signal with 16 × 16 input sizes (Table 5). The average accuracy of eight CNN models trained from driver-end signals is 96.54% compared to the 90.97% of the models trained with fan-end signals. This means that the signal from the drive end is more useful than the fan end for fault diagnosis. This may be caused by several reasons such as the sensor quality, sensor locations, environment effects and so on. Next, we evaluate how our improved D-S fusion algorithm helps improve the fault diagnosis performance of our CNN models. Figure 6 shows the experiment results for the individual CNNs and the IDSCNN models with different fusion sources. In Figure 6a, we trained right CNN models with parameter settings in Table 2 for each load condition (Datasets A, B, and C) with input size of 16 × 16 and test them on all three test sets A, B, and C. We then measure the minimal, maximal, and average fault prediction performances along with the performance by the IDS fusion model that takes the output of the eight models and use the improved IDS fusion algorithm to make prediction. The result at the last row of Figure 6a shows that fusion model ids-de-16 achieves the highest average performance than the maximum performance of individual CNNs. This is still true for the ids-de-32 model in Figure 6c. For the fusion models trained with the fan-end sensor, which has lower diagnosis quality, the fusion model achieves better performance than the average performance of the 16 models. Their performances are also higher than those of individual CNN models for most test scenarios. 

For further analysis, we applied our IDS fusion method to all the 16 CNN models at the drive end, all the 16 CNN models at the fan end, and all 32 CNN models at both ends (ids-de-all, ids-fe-all, and ids-all, respectively). In Figure 6e, we have the following observations. First, the fusion models of both fan-end and drive-end have improved the accuracy by about 2%, a significant improvement for this challenging problem. After combining all 32 CNN models through our IDS method, the final diagnosis accuracy based on two sensors can reach 98.92%, which has increased by almost 9.81% from the worst CNN model based on single sensor in Table 5.

**Figure 7:**
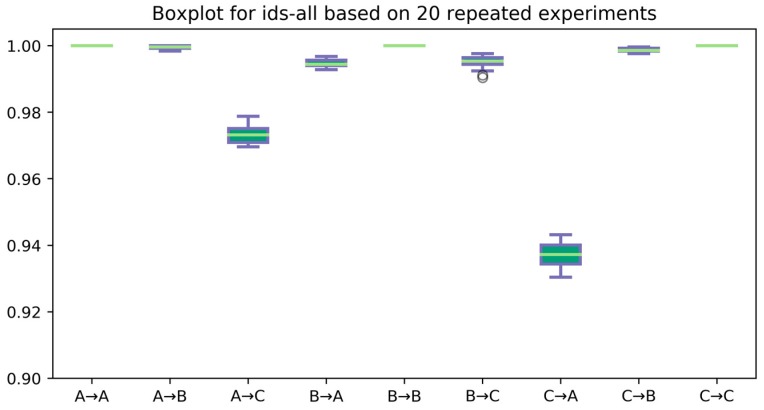
Accuracy of the best IDSCNN models on 20 repeated experiments.

To further validate the robustness of the proposed method, we selected three best IDSCNN models (ids-all), one for each load condition. We then evaluate their performance on 20 randomly sampled test datasets for each load condition and plot the Boxplot in Figure 7. Our first observation is that these IDSCNN models all achieved 100% accuracy for the test datasets of the same load condition, which is rare with the individual CNN models, as shown in Table 5. For test datasets generated from different load conditions, our IDSCNN models achieved an average accuracy higher than 97% with small performance variation for A→B, A→C, B→A, B→C, and C→B, except for the case C→A. It means that the best IDSCNN model trained with signals from load condition C reached an average accuracy of 93% with large variation, which is much lower than the other cases.

**Figure 8:**
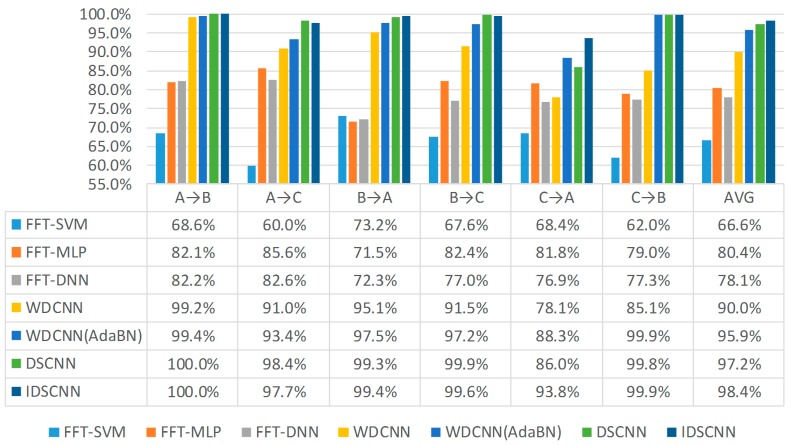
Comparison with other fault diagnosis models.

We compare our best DSCNN (CNN models fused with traditional D-S method) models and the best IDSCNN models with 5 other bearing fault diagnosis models in Figure 8. We can see that the DSCNN method, which combines the CNN models with traditional D-S evidence theory has higher accuracy than FFT-SVM, FFT-MLP, FFT-DNN [48], WDCNN [49] and WDCNN (AdaBN) [49] on all nine conditions but has lower accuracy than the WDCNN (AdaBN) model on C→A. As shown in the last row of Figure 8, our IDSCNN models achieved the best diagnosis results under all six test scenarios when compared with the first five models. Especially, the accuracy of C→A has been improved from 88.3% for WDCNN (AdaBN) and 86.0% for DSCNN to 93.8%. This can be attributed to the larger number of paradoxical evidences under the C→A diagnosis condition, which the traditional D-S fusion method cannot handle while our improved IDS fusion algorithm can. The diagnosis evidences under other test conditions may have relatively higher consistence, so the diagnosis accuracies of DSCNN and IDSCNN are very similar.

**Figure 9:**
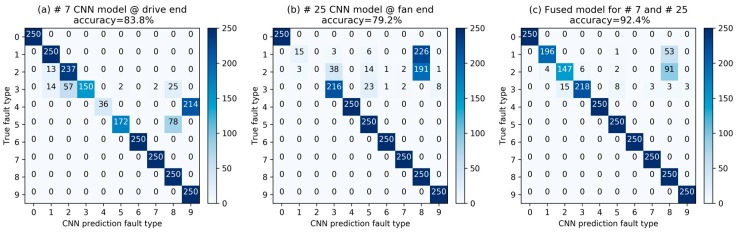
Information fusion between drive-end and fan-end predictions: (**a**) confusion matrix of #7 CNN model trained with drive-end signal (See Table 5) for C→A test; (**b**) confusion matrix of #25 CNN model trained with fan-end signal (See Table 5) for C→A test; and (**c**) confusion matrix of fused model for #7 and #25 for C→A test.

To figure out how our improved D-S fusion algorithm improves the prediction performance, we compared the confusion matrices of a drive-end CNN model, a fan-end CNN model, and the IDSCNN fusion model, as shown in Figure 9. The vertical axis of Figure 9, represents the true labels, while the lateral axis represents the predicted labels. The values in the matrices are the number of predicted samples for each fault type. In Figure 9a, we found that CNN model #7 trained on Dataset C with driver-end signal and tested on test Dataset A performs well except a large number of misclassifications of Type 3, type4 and Type 5 samples. Its total accuracy is 83.8%. On the other hand, Figure 9b shows that CNN model #25 trained on Dataset C with fan-end signal and tested on test Dataset A performs well except significant number of misclassifications of Type 1 and Type 2 samples. Overall, it has an accuracy of 79.2%. These two models have complementary fault classification capabilities, which are exploited by the IDSCNN model. Figure 9c shows that the fusion model achieved the highest performance among the three. The total diagnosis accuracy has improved from 83.8% of the drive-end CNN model, 79.2% of the fan-end CNN model to 92.4% after information fusion.

**Figure 10:**
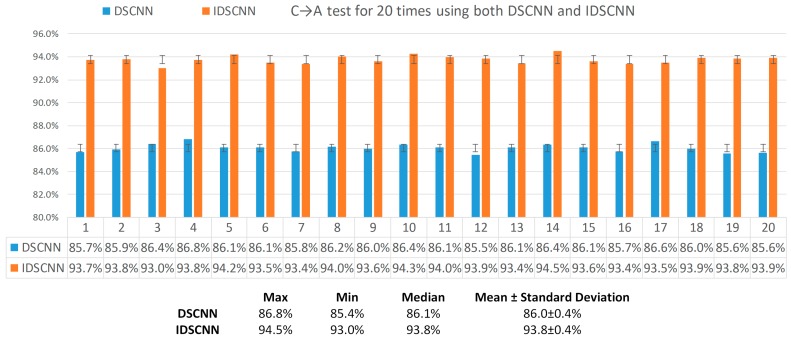
Performance comparison of DSCNN vs IDSCNN on C→A test.

To validate that the performance difference between DSCNN and IDSCNN is statistically significant, we repeated C→A test for twenty times. The DSCNN models and IDSCNN models were trained respectively with samples from the load condition C (Dataset C) and each test datum is randomly sampled under the load condition A (Dataset A). We calculated five statistical parameters (max, min, median, mean and standard deviation) according to the 20 times results of the DSCNN and IDSCNN models. In Figure 10, we can find that the best performance of DSCNN is 86.8% while the worst performance of IDSCNN is 93.0%. The average classification accuracy of the DSCNN models is 86.0 ± 0.4% while the IDSCNN models can classify 93.8 ± 0.4% of the testing data correctly. Though both DSCNN and IDSCNN methods have the same standard deviation 0.4%, it is apparent in Figure 10 that the classification accuracy of the proposed IDSCNN model is higher than that of DSCNN model on C→A test with an average 7.8% improvement.

## 4. Conclusions

This paper presents IDSCNN, an ensemble convolutional neural network model with improved D-S evidence fusion for bearing fault diagnosis. We proposed an improved fusion algorithm based on the traditional D-S evidence theory by rectifying the raw evidence through evidence credibility, which helps to address its paradox evidence issues. Our extensive experiments on the Case Western Reserve University (CWRU) bearing datasets showed that the traditional D-S fusion method fails to combine the evidences/signals from two sensors located at the fan-end and drive-end of the testing rig due to evidence conflicts. On the other hand, our IDSCNN model can deal with four common paradoxical evidences and achieved higher diagnosis accuracy by fusing signal from two sensors when compared with SVM, MLP, DNN, WDCNN, WDCNN (AdaBN) and DSCNN models. Our IDSCNN model has shown good adaptability on the CWRU bearing fault datasets under different load conditions, which makes it suitable for high-performance bearing fault diagnosis under varying load conditions with multi-sensors signals.

## Figures and Tables

**Table 1:** BPAs for four common paradoxes.

Paradoxes	Evidences	Propositions				
		*A*	*B*	*C*	*D*	*E*
Complete conflict paradox	*m*_1_	1	0	0	-	-
	*m*_2_	0	1	0	-	-
	*m*_3_	0.8	0.1	0.1	-	-
	*m*_4_	0.5	0.2	0.3	-	-
0 trust paradox	*m*_1_	0.5	0.2	0.3	-	-
	*m*_2_	0.5	0.2	0.3	-	-
	*m*_3_	0	0.9	0.1	-	-
	*m*_4_	0.5	0	0.3	-	-
1 trust paradox	*m*_1_	0.9	0.1	0	-	-
	*m*_2_	0	0.1	0.9	-	-
	*m*_3_	0.1	0.15	0.75	-	-
	*m*_4_	0.1	0.15	0.75	-	-
High conflict paradox	*m*_1_	0.7	0.1	0.1	0	0.1
	*m*_2_	0	0.5	0.2	0.1	0.2
	*m*_3_	0.6	0.1	0.15	0	0.15
	*m*_4_	0.55	0.1	0.1	0.15	0.1
	*m*_5_	0.6	0.1	0.2	0	0.1

**Table 2:** Structures and parameters of the proposed CNNs.

Parameter Setting					
No	Input	C1	C2	C3	Stride
1	16 × 16 × 1	[5,5,1,10]	[4,4,10,16]	[4,4,16,12]	(1,2,1)
2	16 × 16 × 1	[5,5,1,10]	[4,4,10,16]	[4,4,16,16]	(1,2,1)
3	16 × 16 × 1	[5,5,1,10]	[4,4,10,20]	[4,4,16,12]	(1,2,1)
4	16 × 16 × 1	[5,5,1,10]	[4,4,10,20]	[4,4,16,16]	(1,2,1)
5	16 × 16 × 1	[5,5,1,10]	[4,4,10,16]	[4,4,16,12]	(1,2,2)
6	16 × 16 × 1	[5,5,1,10]	[4,4,10,16]	[4,4,16,16]	(1,2,2)
7	16 × 16 × 1	[5,5,1,10]	[4,4,10,20]	[4,4,16,12]	(1,2,2)
8	16 × 16 × 1	[5,5,1,10]	[4,4,10,20]	[4,4,16,16]	(1,2,2)
9	32 × 32 × 1	[5,5,1,10]	[4,4,10,16]	[4,4,16,12]	(1,2,1)
10	32 × 32 × 1	[5,5,1,10]	[4,4,10,16]	[4,4,16,16]	(1,2,1)
11	32 × 32 × 1	[5,5,1,10]	[4,4,10,20]	[4,4,16,12]	(1,2,1)
12	32 × 32 × 1	[5,5,1,10]	[4,4,10,20]	[4,4,16,16]	(1,2,1)
13	32 × 32 × 1	[5,5,1,10]	[4,4,10,16]	[4,4,16,12]	(1,2,2)
14	32 × 32 × 1	[5,5,1,10]	[4,4,10,16]	[4,4,16,16]	(1,2,2)
15	32 × 32 × 1	[5,5,1,10]	[4,4,10,20]	[4,4,16,12]	(1,2,2)
16	32 × 32 × 1	[5,5,1,10]	[4,4,10,20]	[4,4,16,16]	(1,2,2)

**Table 3:** Description of rolling element bearing datasets.

Fault Location		None	Ball			Inner Race			Outer Race		
Fault Type		0	1	2	3	4	5	6	7	8	9
Fault Diameter (inch)		0	0.007	0.014	0.021	0.007	0.014	0.021	0.007	0.014	0.021
Dataset A	Train	1000	1000	1000	1000	1000	1000	1000	1000	1000	1000
	Test	250	250	250	250	250	250	250	250	250	250
Dataset B	Train	1000	1000	1000	1000	1000	1000	1000	1000	1000	1000
	Test	250	250	250	250	250	250	250	250	250	250
Dataset C	Train	1000	1000	1000	1000	1000	1000	1000	1000	1000	1000
	Test	250	250	250	250	250	250	250	250	250	250

**Table 4:** Comparison of the synthesis results.

Paradoxes	Methods	Propositions					
		*A*	*B*	*C*	*D*	*E*	*Θ*
Complete conflict paradox (*k* = 1)	Yager	0	0	0	-	-	1
	Sun	0.0917	0.0423	0.0071	-	-	0.8589
	Murphy	0.8204	0.1748	0.0048	-	-	0.0000
	Deng	0.8166	0.1164	0.0670	-	-	0.0000
	IDS	**0.9284**	0.0716	0.0000	-	-	
0 trust paradox (*k* = 0.99)	Yager	0.0000	0.7273	0.2727	-	-	0.0000
	Sun	0.0525	0.0597	0.0377	-	-	0.8501
	Murphy	0.4091	0.4091	0.1818	-	-	0.0000
	Deng	0.4318	0.2955	0.2727	-	-	0.0000
	IDS	**0.7418**	0.2582	0.0000	-	-	0.0000
1 trust paradox (*k* = 0.99)	Yager	0.0000	1.0000	0.0000	-	-	0.0000
	Sun	0.0388	0.0179	0.0846	-	-	0.8587
	Murphy	0.1676	0.0346	0.7978	-	-	0.0000
	Deng	0.1388	0.1318	0.7294	-	-	0.0000
	IDS	0.0594	0.0000	**0.9406**	-	-	0.0000
High conflict paradox (*k* = 0.9999)	Yager	0.0000	0.3571	0.4286	0.0000	0.2143	0.0000
	Sun	0.0443	0.0163	0.0136	0.0045	0.0118	0.9094
	Murphy	**0.7637**	0.1031	0.0716	0.0080	0.0538	0.0000
	Deng	0.5324	0.1521	0.1462	0.0451	0.1241	0.0000
	IDS	0.6210	0.1456	0.1308	0.0000	0.1026	0.0000

**Table 5:** Results of the 32 CNN Models.

Different Combinations Among Training Data Set and Testing Data Set												
**Training Data Set**		A			B			C				
**Testing Data Set**		A	B	C	A	B	C	A	B	C		
**Expression**		A→A	A→B	A→C	B→A	B→B	B→C	C→A	C→B	C→C		
**Note:** In Table 5 and subsequent contents, A, B and C stand for three different datasets. The first letter stands for the training dataset, the second letter stands for the testing dataset.												
**Example:** A→B means we trained our models on Dataset A and tested our models on Dataset B.												
**Drive End with 16 × 16 × 1 Input**												
**CNN No.**	**Model No.**	**A**→**A**	**A**→**B**	**A**→**C**	**B**→**A**	**B**→**B**	**B**→**C**	**C**→**A**	**C**→**B**	**C**→**C**	**AVG**	**AVG2**
1	1	99.28%	98.71%	87.60%	97.09%	99.85%	96.58%	86.63%	98.90%	99.92%	96.06%	96.54%
2	2	99.93%	99.57%	95.67%	99.35%	99.87%	97.43%	89.81%	99.39%	100.00%	97.89%	
3	3	99.69%	99.42%	90.91%	95.93%	99.82%	95.94%	81.55%	98.54%	99.82%	95.74%	
4	4	99.81%	98.80%	86.44%	98.35%	99.81%	95.58%	94.65%	99.57%	99.90%	96.99%	
5	5	99.86%	99.54%	94.18%	98.17%	99.97%	99.05%	88.07%	99.04%	99.86%	97.53%	
6	6	99.69%	99.41%	95.46%	97.12%	99.91%	96.44%	88.39%	98.66%	99.90%	97.22%	
7	7	99.78%	99.40%	90.92%	98.33%	99.95%	97.90%	82.41%	92.53%	99.86%	95.68%	
8	8	99.50%	99.35%	92.52%	95.99%	99.59%	94.26%	84.12%	91.86%	99.89%	95.23%	
**Drive End with 32 × 32 × 1 Input**												
**CNN No.**	**Model No.**	**A→A**	**A→B**	**A→C**	**B→A**	**B→B**	**B→C**	**C→A**	**C→B**	**C→C**	**AVG**	**AVG2**
9	9	99.98%	99.38%	93.59%	98.34%	100.00%	92.47%	93.49%	99.53%	99.98%	97.42%	96.46%
10	10	99.57%	95.66%	85.17%	96.17%	99.81%	91.90%	85.56%	96.97%	99.87%	94.52%	
11	11	99.99%	99.10%	86.93%	97.34%	99.98%	90.16%	91.14%	98.78%	99.77%	95.91%	
12	12	99.62%	97.43%	92.38%	98.47%	99.78%	99.02%	88.15%	97.85%	99.92%	96.96%	
13	13	100.00%	99.74%	93.32%	97.73%	99.98%	94.36%	91.77%	99.59%	99.92%	97.38%	
14	14	99.82%	97.97%	90.40%	95.85%	99.81%	95.74%	89.37%	99.26%	99.93%	96.46%	
15	15	99.96%	99.00%	93.90%	98.16%	99.99%	98.63%	80.45%	90.76%	99.70%	95.62%	
16	16	99.99%	99.30%	92.96%	98.57%	99.91%	98.94%	88.46%	98.62%	99.90%	97.40%	
**Fan End with 16 × 16 × 1 Input**												
**CNN No.**	**Model No.**	**A→A**	**A→B**	**A→C**	**B→A**	**B→B**	**B→C**	**C→A**	**C→B**	**C→C**	**AVG**	**AVG2**
1	17	99.77%	97.12%	80.11%	93.94%	99.83%	93.27%	67.51%	84.13%	99.81%	90.61%	90.97%
2	18	99.98%	98.31%	77.67%	92.76%	99.93%	94.98%	71.07%	83.27%	99.97%	90.88%	
3	19	99.95%	98.29%	80.28%	94.87%	99.94%	94.90%	70.33%	79.86%	99.93%	90.93%	
4	20	99.38%	94.03%	79.16%	92.70%	99.80%	96.87%	73.21%	86.88%	99.97%	91.33%	
5	21	100.00%	98.37%	77.61%	94.70%	99.98%	97.68%	70.17%	81.43%	99.97%	91.10%	
6	22	99.96%	97.47%	76.24%	93.46%	99.96%	96.78%	78.89%	84.22%	100.00%	91.89%	
7	23	99.59%	95.96%	78.26%	95.77%	99.88%	90.39%	73.15%	82.76%	99.94%	90.63%	
8	24	99.88%	97.19%	73.65%	91.35%	99.79%	96.37%	72.27%	82.88%	99.98%	90.37%	
**Fan End with 32 × 32 × 1 Input**												
**CNN No.**	**Model No.**	**A→A**	**A→B**	**A→C**	**B→A**	**B→B**	**B→C**	**C→A**	**C→B**	**C→C**	**AVG**	**AVG2**
9	25	100.00%	98.37%	84.52%	88.38%	99.72%	83.03%	79.83%	94.69%	100.00%	92.06%	90.37%
10	26	99.92%	96.87%	78.06%	88.89%	99.80%	90.12%	72.68%	83.65%	99.94%	89.99%	
11	27	99.99%	96.76%	73.35%	91.93%	99.99%	86.39%	71.95%	81.65%	99.97%	89.11%	
12	28	99.93%	94.40%	79.80%	88.93%	99.92%	89.74%	76.31%	83.89%	99.96%	90.32%	
13	29	99.99%	97.61%	78.72%	91.32%	99.99%	88.84%	74.38%	83.33%	99.99%	90.46%	
14	30	99.96%	96.83%	76.55%	89.38%	99.97%	88.25%	71.00%	81.10%	99.98%	89.22%	
15	31	99.99%	98.48%	83.78%	89.70%	99.98%	84.92%	76.65%	84.68%	99.98%	90.91%	
16	32	99.99%	98.28%	83.21%	89.11%	100.00%	90.30%	72.47%	84.43%	99.97%	90.86%	

## References

[1] Gelman L., Murray B., Patel T.H., Thomson A. (2014). Vibration diagnostics of rolling bearings by novel nonlinear non-stationary wavelet bicoherence technology. Eng. Struct..

[2] Jena D.P., Panigrahi S.N. (2015). Automatic gear and bearing fault localization using vibration and acoustic signals. Appl. Acoust..

[3] Peng E.G., Liu Z.L., Zhou X.C., Zhao M.Y., Lan F. (2011). Application of Vibration and Noise Analysis in Water-Lubricated Rubber Bearings Fault Diagnosis. Adv. Mater. Res..

[4] Janssens O., Schulz R., Slavkovikj V., Stockman K., Loccufier M., Walle R.V.D., Hoecke S.V. (2015). Thermal image based fault diagnosis for rotating machinery. Inf. Phys. Technol..

[5] Chen J., Zi Y., He Z., Wang X. (2013). Adaptive redundant multiwavelet denoising with improved neighboring coefficients for gearbox fault detection. Mech. Syst. Signal Proc..

[6] Safin N.R., Prakht V.A., Dmitrievskii V.A., Dmitrievskii A.A. (2014). Diagnosis of Bearing Faults of Induction Motors by Spectral Analysis of Stator Currents. Adv. Mater. Res..

[7] Yang J., Zhang J., Zhang G., Liu Y. (2013). Fault Diagnosis of Transmission Rolling Bearing Based on Wavelet Analysis and Binary Tree Support Vector Machine. Int. J. Digit. Content Technol. Its Appl..

[8] Mohanty S., Gupta K.K., Raju K.S., Singh A., Snigdha S. Vibro acoustic signal analysis in fault finding of bearing using Empirical Mode Decomposition. Prceedings of the International Conference on Advanced Electronic Systems.

[9] Luo J., Yu D., Liang M. (2013). A kurtosis-guided adaptive demodulation technique for bearing fault detection based on tunable-Q wavelet transform. Meas. Sci. Technol..

[10] Cui L., Wu N., Ma C., Wang H. (2016). Quantitative fault analysis of roller bearings based on a novel matching pursuit method with a new step-impulse dictionary. Mech. Syst. Signal Proc..

[11] Misra M., Yue H.H., Qin S.J., Ling C. (2002). Multivariate process monitoring and fault diagnosis by multi-scale PCA. Comput. Chem. Eng..

[12] Widodo A., Yang B.S. (2007). Application of nonlinear feature extraction and support vector machines for fault diagnosis of induction motors. Exp. Syst. Appl..

[13] Muralidharan A., Sugumaran V., Soman K.P. (2016). Bearing Fault Diagnosis Using Vibration Signals by Variational Mode Decomposition and Naïve Bayes Classifier. Int. J. Robot. Autom..

[14] Pandya D.H., Upadhyay S.H., Harsha S.P. (2013). Fault diagnosis of rolling element bearing with intrinsic mode function of acoustic emission data using APF-KNN. Exp. Syst. Appl..

[15] Hajnayeb A., Ghasemloonia A., Khadem S.E., Moradi M.H. (2011). Application and comparison of an ANN-based feature selection method and the genetic algorithm in gearbox fault diagnosis. Exp. Syst. Appl..

[16] FernáNdez-Francos D., MartíNez-Rego D., Fontenla-Romero O., Alonso-Betanzos A. (2013). Automatic bearing fault diagnosis based on one-class *v*-SVM. Comput. Ind. Eng..

[17] Jia G., Yuan S., Tang C., Xiong J. Fault diagnosis of roller bearing using feedback EMD and decision tree. Prceedings of the International Conference on Electric Information and Control Engineering.

[18] Peng H.W., Chiang P.J. Control of mechatronics systems: Ball bearing fault diagnosis using machine learning techniques. Prceedings of the 8th Asian Control Conference.

[19] Jedliński Ł., Jonak J. (2015). Early fault detection in gearboxes based on support vector machines and multilayer perceptron with a continuous wavelet transform. Appl. Soft Comput..

[20] Kankar P.K., Sharma S.C., Harsha S.P. (2013). Fault diagnosis of rolling element bearing using cyclic autocorrelation and wavelet transform. Neurocomputing.

[21] Zhang X., Qiu D., Chen F. (2015). Support vector machine with parameter optimization by a novel hybrid method and its application to fault diagnosis. Neurocomputing.

[22] Ji N.N., Zhang J.S., Zhang C.X. (2014). A sparse-response deep belief network based on rate distortion theory. Pattern Recognit..

[23] Leng B., Zhang X., Yao M., Xiong Z. (2015). A 3D model recognition mechanism based on deep Boltzmann machines. Neurocomputing.

[24] Katayama K., Ando M., Horiguchi T. (2003). Model of MT and MST areas using an autoencoder. Physics A.

[25] Zhang W., Li R., Deng H., Li W., Lin W., Ji S., Shen D. (2015). Deep Convolutional Neural Networks for Multi-Modality Isointense Infant Brain Image Segmentation. Neuroimage.

[26] Guo X., Chen L., Shen C. (2016). Hierarchical adaptive deep convolution neural network and its application to bearing fault diagnosis. Measurement.

[27] Chen Z.Q., Li C., Sanchez R.V. (2015). Gearbox Fault Identification and Classification with Convolutional Neural Networks. Shock Vib..

[28] Ince T., Kiranyaz S., Eren L., Askar M., Gabbouj M. (2016). Real-time motor fault detection by 1-d convolutional neural networks. IEEE Trans. Ind. Electron..

[29] Tran V.T., Althobiani F., Ball A. (2014). An approach to fault diagnosis of reciprocating compressor valves using Teager–Kaiser energy operator and deep belief networks. Exp. Syst. Appl..

[30] Yager R.R. (1987). On the Dempster-Shafer framework and new combination rules. Inf. Sci..

[31] Hégarat-Mascle S.L., Bloch I., Vidal-Madjar D. (1997). Application of Dempster-Shafer evidence theory to unsupervised classification in multisource remote sensing. Geosci. Remote Sens..

[32] Li Y.-B., Wang N., Zhou C. Based on D-S evidence theory of information fusion improved method. Prceedings of the International Conference on Computer Application and System Modeling.

[33] Dou Z., Xu X., Lin Y., Zhou R. (2014). Application of D-S Evidence Fusion Method in the Fault Detection of Temperature Sensor. Math. Probl. Eng..

[34] Hui K.H., Meng H.L., Leong M.S., Al-Obaidi S.M. (2017). Dempster-Shafer evidence theory for multi-bearing faults diagnosis. Eng. Appl. Artif. Intell..

[35] Browne F., Rooney N., Liu W., Bell D. (2013). Integrating textual analysis and evidential reasoning for decision making in engineering design. Knowl. Based Syst..

[36] Avci E. (2013). A new method for expert target recognition system: Genetic wavelet extreme learning machine (GAWELM). Exp. Syst. Appl..

[37] Dong G., Kuang G. (2015). Target Recognition via Information Aggregation Through Dempster–Shafer’s Evidence Theory. IEEE Geosci. Remote Sens. Lett..

[38] Xing H., Hua G., Han Y., Liu C., Dang Y. (2016). Quantitative MMM evaluation of weld levels based on information entropy and DS evidence theory. Chin. J. Sci. Instrum..

[39] Kang J., Gu Y.B., Li Y.B. (2012). Multi-sensor information fusion algorithm based on DS evidence theory. J. Chin. Inert. Technol..

[40] Li Y., Chen J., Ye F., Liu D. (2015). The Improvement of DS Evidence Theory and Its Application in IR/MMW Target Recognition. J. Sens..

[41] AbuMahfouz I.A. (2005). A comparative study of three artificial neural networks for the detection and classification of gear faults. Int. J. Gen. Syst..

[42] Smith W.A., Randall R.B. (2015). Rolling element bearing diagnostics using the Case Western Reserve University data: A benchmark study. Mech. Syst. Signal Proc..

[43] Case Western Reserve University Bearing Data Center Website. http://csegroups.case.edu/bearingdatacenter/home.

[44] Yager R.R. (2002). On the aggregation of prioritized belief structures. IEEE Trans. Syst. Man Cybern. Part A.

[45] Sun Q., Ye X., Gu W. (2000). A New Combination Rules of Evidence Theory. Acta Electron. Sin..

[46] Murphy C.K. (2000). Combining belief functions when evidence conflicts. Decis. Support Syst..

[47] Deng Y., Shi W.K., Zhu Z.F. (2004). Efficient combination approach of conflict evidence. J. Infrared Millim. Waves.

[48] Jia F., Lei Y., Lin J., Zhou X., Lu N. (2016). Deep neural networks: A promising tool for fault characteristic mining and intelligent diagnosis of rotating machinery with massive data. Mech. Syst. Signal Proc..

[49] Zhang W., Peng G., Li C., Chen Y., Zhang Z. (2017). A New Deep Learning Model for Fault Diagnosis with Good Anti-Noise and Domain Adaptation Ability on Raw Vibration Signals. Sensors.

